# Prescribing patterns and preferences for moderate-to-severe pediatric (<12 years of age) atopic dermatitis: A survey of Canadian practitioners

**DOI:** 10.1016/j.jdin.2023.10.003

**Published:** 2023-10-28

**Authors:** Michele L. Ramien, Vipul Jain, James Bergman, Danielle Marcoux, Irene Lara-Corrales

**Affiliations:** a Department of Pediatrics, University of Calgary, Alberta Children’s Hospital, Calgary, Alberta, Canada; b Division of Dermatology, Department of Medicine, University of Calgary, Calgary, Alberta, Canada; c Division of Clinical Immunology and Allergy, Department of Internal Medicine, Michael G. DeGroote School of Medicine, McMaster University, Hamilton, Ontario, Canada; d Probity Medical Research, Niagara Falls, Ontario, Canada; e Allergy Research Canada Inc, Niagara Falls, Ontario, Canada; f Department of Dermatology and Skin Science, University of British Columbia, Vancouver, British Columbia, Canada; g Division of Dermatology, Department of Pediatrics, Sainte-Justine University Hospital Center, University of Montreal, Montreal, Quebec, Canada; h The Hospital for Sick Children and Department of Pediatrics, University of Toronto, Toronto, Ontario, Canada

**Keywords:** atopic dermatitis, biologics, Canada/Canadian, eczema, pediatric dermatology, survey study, therapeutics

*To the Editor:* Approximately 15% of Canadians aged 0.5 to 17 years have atopic dermatitis (AD), which significantly impairs quality of life, with a burden that increases with increasing severity of AD.^1^, ^2^, ^3^, ^4^ Despite its burden for both children and their families, recent Canadian data indicate that only one-third of pediatric AD patients receive systemic therapy to control their AD, suggesting a pattern of undertreatment and the need to explore barriers.^3^^,^^5^

Five Canadian clinicians experienced in AD management developed a 23-question survey to explore current prescriber preferences and patterns for pediatric AD (patients ≤12 years old with moderate-to-severe disease). A list of all clinicians identified as practicing pediatric dermatology in Canada was assembled (40); each of these clinicians received an email inviting them to participate in the survey. Twenty-one dermatologists and pediatric dermatologists agreed to receive the survey, and 15 completed it (71% completion rate, Survey Monkey platform). Responses were anonymous. This study received approval from The SickKids Quality Improvement Research Ethics Board (QIP-2022-09-30T11-04-05).

Most survey respondents practiced in academic centers (80%) and had been in practice for 5 years or longer (80%) as dermatologists or pediatric dermatologists (93%). Almost three-quarters of respondents saw more than 50 patients with AD per month; all respondents saw at least 10 patients with AD per month (Table I).

**Table I:** Survey respondent and pediatric atopic dermatitis population characteristics

Question	Responses	Frequency, *n* (%)
Which one of the following best describes your primary practice type (>50% of your clinical time)?	Academic hospital	12 (80.0)
	Academic community practice	1 (6.7)
	Community private practice	2 (13.3)
How many years have you been in practice?	<5 y	3 (20.0)
	5-10 y	3 (20.0)
	11-15 y	2 (13.3)
	16-30 y	5 (33.3)
	>30 y	2 (13.3)
What is your specialty?	Dermatology	5 (33.3)
	Pediatric dermatology	9 (60.0)
	Pediatrics	1 (6.7)
	Allergy/immunology	0 (0)
How many patients with atopic dermatitis do you see in an average month?	<10	0 (0%)
	>10 and <50	4 (26.7)
	>50 and <75	4 (26.7)
	>75 and <100	4 (26.7)
	>100	3 (20.0)
What percentage of your moderate-to-severe patients with AD are?∗	Infants (2 mo-2 y):	26
	Preschool children (3-5 y):	26
	Children (6-11 y):	27
	Adolescent (12 y-18 y):	17
How do you define “moderate-to-severe AD” in your patient population (check all that apply)?	Gestalt	11 (73.3)
	Validated tools/instruments	12 (80.0)
	Patient-reported outcomes	9 (60.0)
	Other (please specify)†	3 (20.0)

*AD*, Atopic dermatitis.

∗ Two respondents’ selections did not add to 100%, presumably because they also treat adults.

† “Other” responses specified included: “clinical opinion"; "impact on QoL, level of pruritus, level of medications utilized"; and "gestalt taking into account body surface involvement, number of skin infections, presence of lichenification, and impact on quality of life.”

The AD population treated by respondents consisted of roughly equal proportions of infants, preschool children, and children, with fewer adolescents (Table I). Moderate-to-severe AD, diagnosed on gestalt or using validated instruments (Table I), accounted for 67% of infant patients and 73% of preschool patients (Table II).

**Table II:** Treated population severity and prior therapies

Question	Infants (2 mo-2 y) Mean, %	Preschool children (3 y- 5 y) Mean, %	Children (6 y-11 y) Mean, %
What percentage of mild, moderate, or severe patients with AD in the following age group do you have in your practice? (*n* = 14)			
Mild (IGA/PGA = 1 or 2)	33.2	26.3	24.5
Moderate (IGA/PGA = 3)	45.0	47.3	48.9
Severe (IGA/PGA = 4)	22.1	26.1	25.9
Please indicate the percentage of your moderate-to-severe patients with AD that use the following therapies at any point in the course of their disease management:			
Phototherapy	0	2.3	5.3
Prednisone/prednisolone	3.3	4.7	5.4
Other systemic therapies (cyclosporine, methotrexate, azathioprine, mycophenolate mofetil, IVIG, etc.)	9.7	14.8	19.5
Biologics (ie, dupilumab, tralokinumab)	3.1	17.3	32.5
JAKi (abrocitinib, upadacitinib)	0	1.7	2.7

*AD*, Atopic dermatitis; *IGA*, investigator’s global assessment; *IVIG*, intravenous immunoglobulins; *JAKi*, Janus kinase inhibitor; *PGA*, physician’s global assessment.

Therapies prescribed at any point for management of moderate-to-severe AD included traditional systemic immunosuppressive agents in 10% and 15% of infant and preschool patients respectively, biologics in 3% of infant and 17% of preschool patients, and Janus kinase inhibitors in 2% of preschool patients. Janus kinase inhibitors were not prescribed to infants.

Nearly all respondents (93%) believed that infants and children with moderate-to-severe AD are currently being undertreated compared to adolescents and adults, citing: hesitancy around the use of topical corticosteroids, lack of access to safe, indicated, and efficacious treatments, provider hesitancy over systemic therapy safety concerns, and insufficient health care provider education about moderate-to-severe AD. Additional barriers to treatment of AD in infants and children cited by respondents included corticophobia, provider time, logistical challenges (ie, injections, phototherapy), and administrative burden.

Clinical concerns about using systemic therapies endorsed by respondents include lack of an indication for pediatric AD and safety concerns including the use of live vaccines and long-term monitoring for adverse effects.

Our survey results highlight an underrecognized group of Canadian patients with AD (≤5 years old with moderate-to-severe AD) whose need for treatment remains a practice gap. The limitations of our survey include small sample size and limited scope. Safety and efficacy remain key concerns for providers caring for this population but treatment options are limited. Barriers to systemic therapy for very young patients with AD include the low number of experienced providers, provider concerns about adverse effects, and medication accessibility.

## Conflicts of interest

Dr Ramien has been an advisor/consultant for, has received grants/honoraria from, and/or has been a speaker for AbbVie, Boehringer Ingelheim, Eli Lilly, LEO Pharma, Pfizer, and Sanofi. Dr Jain has received honoraria for grants and/or research funding, for being a speaker, consultant, advisory board member, and/or investigator for the following companies: Pediapharm, Aralez, Sanofi, Regeneron Pharmaceuticals, Bausch, Novartis, Medexus, ALK, Celgene, LEO, Mylan, Amgen, AbbVie, Pfizer, Eli Lilly, Galderma, AstraZeneca, Jenssen, Arcutis, Kymab, and GSK. Dr Bergman has been a consultant for AbbVie, Apogee, Aralez, Bausch, Cipher, Galderma, Johnson & Johnson, La Roche Posay, LEO, L’Oreal, Nestle, Novartis, Pierre Fabre, Pfizer, and Sanofi; has been a speaker for AbbVie, Aralez, Bausch, Galderma, Johnson & Johnson, La Roche Posay, LEO, L’Oreal, Pierre Fabre, Pfizer, and Sanofi; and has served on the Board of Directors for the Eczema Society of Canada and Camp Liberte. Dr Marcoux has been an investigator, consultant, and/or speaker for AbbVie, LEO Pharma, Lilly, Novartis, Pfizer, Sanofi, and Regeneron. Dr Lara-Corrales has received grants/research support from AbbVie, Janssen, Clementia, Eli Lilly, Timber, Arcutis, DeBRA Canada, EB Medical Research Foundation, EB Research Partnership, Canadian Dermatology Foundation, International Psoriasis Council, CANAAF, and Physicians Services Incorporated (PSI) Foundation; has been a consultant for Janssen, Sanofi, Abeona, Johnson & Johnson, Avicanna, Ipsen, Novartis, and Eli Lilly; and a speaker for Sanofi and AbbVie.
